# Editorial: Intersection of Hormones and Neuropeptides in the Brain

**DOI:** 10.3389/fnbeh.2022.886591

**Published:** 2022-04-22

**Authors:** Susan L. Zup, Jin Ho Park, Juan M. Dominguez

**Affiliations:** ^1^Developmental and Brain Sciences Program (DBS), Department of Psychology, University of Massachusetts Boston, Boston, MA, United States; ^2^Departments of Psychology, The University of Texas at Austin, Austin, TX, United States; ^3^Department of Pharmacology & Toxicology, The University of Texas at Austin, Austin, TX, United States

**Keywords:** neurotransmitters, neuropeptides, hormones, sex differences, cognition, neurodevelopment

Many compounds beyond “classic neurotransmitters” exert profound influences on the brain and behavior. Among these, neuropeptides and hormones play a crucial role during the development of the nervous system, in the regulation of behaviors, and in the etiologies of behavioral pathologies or disorders.

“These chemical messengers, however, or “hormones” (from óρμ$\overset{´}{\alpha }$ω, I excite or arouse), as we might call them, have to be carried from the organ where they are produced to the organ which they affect by means of the blood stream and the continually recurring physiological needs of the organism must determine their repeated production and circulation through the body” (Starling, 1905). It was with this statement that, in 1905, renowned British physiologist Ernest Starling gave birth to the concept of “hormones” as we know it today, when describing “substances produced for effecting the correlation of organs within the body, through the intermediation of the blood stream” (Starling, 1905). Our definition of hormones has not changed but by little since 1905, which is to say there are now several more hormones beyond secretin and includes a variety of chemical messengers secreted from endocrine glands directly into the blood stream relaying signals to other organs where they exert some influence on the recipient organ's physiology.

While some similarities exist, hormones are different from neuropeptides, which are small chain amino acids synthesized and secreted by neurons or neurosecretory cells that act as signaling molecules to nearby recipient cells. Our appreciation of neuropeptides arose, in part, from seminal work by Victor Mutt (1923–1998), Berta Scharrer (1906–1995), and Ernst Scharrer (1905–1965), among other early pioneers, who elucidated the fundamental importance of neurosecretion and peptides for the regulation of physiological functions. Following on the work of these early pioneers, the late 1960s and early 1970s brought a wave of discoveries revealing how peptides affect neurobehavioral function. Much of this built on David de Wied's (1925–2004) groundbreaking discoveries demonstrating the important role of peptides as signaling molecules in the nervous system, particularly his research into the influence of peptide hormones adrenocorticotropin releasing hormone (ACTH), melanocyte-stimulating hormone (MSH), and vasopressin on brain function and behavior (Gispen, 2010). Ongoing advances in neuroscience techniques, as well as novel questions posed to uncover the mechanism of interaction at the molecular, cellular, and genetic level continue to expand our understanding of neuropeptides and their role in neural function.

To date, too many neuropeptides have been discovered to list in this brief introduction, however, some of the more commonly recognized include vasopressin, cholecystokinin (CCK), substance P, β-endorphins, prolactin, enkephalins, galanin, kisspeptins, and oxytocin. While this is by no means an exhaustive list, it provides insight into the diversity of peptides that have been discovered and the wide array of neurophysiological functions that they regulate. Consider, for example, the central role of vasopressin and oxytocin in the regulation of complex social cognition and social behaviors (e.g., Meyer-Lindenberg et al., 2011), the role of CCK and galanin in the regulation of feeding behaviors (Lee et al., 1994; Ritter et al., 1999), or the role of prolactin and kisspeptin in the regulation of maternal and sexual behaviors (Erskine, 1995; Kruger et al., 2002; Smith et al., 2006; Kauffman et al., 2007).

The specific interaction of neuropeptides and hormones, with or without input from classic neurotransmitters, alters basic neural functions as well as behavioral outcomes, thus expanding the repertoire of responses through which the brain can react to environmental cues. Consider as a classic example of this interaction the regulation of corticotropin-releasing hormone (CRH), which is important for various physiological functions and for the central regulation of numerous behaviors, particularly stress and stress response. Increased production of CRH induces activation of β-endorphin neurons in the hypothalamus, these β-endorphin neurons innervate CRH neurons to inhibit further CRH release, creating classic negative feedback (Veening and Barendregt, 2015). This special issue is not intended to be a comprehensive treatise on neuropeptides but rather a contribution to what is already a large and continuously growing body of literature highlighting individual hormones and neuropeptides in the brain, in some instances the intersection between the two, and their influence on behaviors [for a detailed analysis of neuropeptides see Strand (1999) or van den Pol (2012), for example].

For as long as there have been studies on sexual behaviors so too has there been an interest in how hormones influence sex. In fact, Arnold Berthold, known for pioneering experiments in endocrinology and who is credited with the first recognized experiment in behavioral endocrinology, pointed to testicular secretions in roosters as important for the expression of sexual activity in males. Berthold found that capons failed to display the stereotypical behaviors normally expected of a rooster, but that these deficits recovered with reimplantation of the testes (Quiring, 1944). Since this early formative work, many others have followed with extensive analyses of how gonadal hormones interact with neurotransmitters to regulate sexual behaviors in a variety of male and female species [e.g., Hull and Dominguez (2015), Pfaus et al. (2015)]. Studies into the idiosyncrasies of this interaction continue to be a topic of great interest to many. In their review article, Glutamate in Male and Female Sexual Behavior: Receptors, Transporters, and Steroid Independence, Chiang and Park provide a comprehensive analysis highlighting the importance of glutamate as a neurotransmitter in the regulation of sexual behaviors in both males and females. They pay particular attention to the potential role of glutamate on steroid-independent sexual behaviors in males and females, while posing several innovative questions that when answered will help us better understand the idiosyncrasies underlying the central regulation of sexual behaviors.

β-endorphins, which are produced primarily in the pituitary gland but also in the hypothalamus and certain cells in the immune system, function through various mechanisms in both the central and peripheral nervous systems to relieve pain when bound to their μ-opioid receptors (Hartwig, 1991). While no cure is yet available, electroacupuncture, as a complementary and alternative therapy, plays a critical role in the management of neuropathic pain associated with brachial plexus root avulsion (BPRA). In their article, Pain Relief Dependent on IL-17-CD4+ T Cell–β-Endorphin Axis in Rat Model of Brachial Plexus Root Avulsion After Electroacupuncture Therapy, Xu et al. examined whether the IL-17-CD4+ T lymphocyte-β-endorphin axis factors into the pain-relieving properties of electroacupuncture following BPRA. They discovered that that BPRA-induced neuropathic pain is relieved by electroacupuncture via IL-17-CD4+ T lymphocyte-β-endorphin mediated analgesic effects. These results showed for the first time that pain caused by BPRA surgery could be significantly relieved by electroacupuncture treatment through modulation of the immune system and the IL-17-CD4+ T cell-β-endorphin axis.

The hypothalamus and pituitary orchestrate regulation of the endocrine system, in part, *via* the hypothalamic-pituitary-adrenal (HPA) and hypothalamic-pituitary-gonadal (HPG) axes. The HPA, in particular, regulates the body's response to factors that threaten homeostasis, namely stress. During acute stress, parvocellular cells in the hypothalamus secrete corticotropin releasing hormone (CRH), which induces synthesis and release of ACTH from the pituitary, this then induces release of mineralocorticoids and glucocorticoids from the adrenal glands. These adrenal hormones impact stress response by acting on a host of downstream mechanisms. In their article, The Hypothalamic-Pituitary-Adrenal Axis: Development, Programming Actions of Hormones, and Maternal-Fetal Interactions, Sheng et al. describe how abnormal development of the HPA axis may result in long-term alterations in neuropeptide and neurotransmitter synthesis in the CNS and glucocorticoid synthesis in the periphery. Interestingly, they examine how the maternal-fetal hypothalamic-pituitary-adrenal axis and disruption of the normal fetal environment may become a major risk factor for neurodevelopmental pathologies in adulthood, including depressive disorders, anxiety, and schizophrenia.

The transition from immature juvenile to reproductively active adult is a critical point for sexually reproducing species and is controlled by the interaction of neuropeptides and hormones (i.e., the HPG axis) in mammals. A similar neurohormonal axis exists in insects which guides the transition from juvenile to adult, prompting Barredo et al. to compare the neuropeptides and steroid hormones underlying both puberty and metamorphosis in their review article, Timing the Juvenile-Adult Neurohormonal Transition: Functions and Evolution. The neurohormonal axes controlling the timing of metamorphosis may be the product of convergent evolution with the mammalian analog. However, Barredo et al. also explore the possibility that key genetic/molecular players within the axes could be present in an earlier ancestor, and that there is a common origin for the neuroendocrine trigger of juvenile-to-adult sexual maturity in both mammals and insects. The genetic and molecular components of the neurohormonal axes in mammals and insects are also implicated in behaviors such as metabolism and sleep, as well as reproductive behaviors. Understanding the intersection of peptides and hormones from a functional and phylogenetic point of view would guide the use of the simpler genetic and cellular circuits in insects as possible model systems.

Acromegaly results from overproduction of growth hormone by the pituitary after normal growth of the skeleton is complete. While not commonly known, depression is a common symptom of acromegaly, which naturally contributes to decreased quality of life for these patients. Whether the related hormonal disturbance that occurs with acromegaly, beyond the physical changes induced by the disease, also play a significant role in the pathophysiology of depression is unclear. To this end, Algahtany et al. examined whether transsphenoidal surgeries relieved depressive symptoms associated with acromegaly. In their article, The Role of Growth Hormone in Depression: A Human Model, they report that transsphenoidal surgery decreased levels of growth hormone and insulin like growth factor-1, this was expected, but interestingly the surgery also decreased depression scores. These findings provide an interesting link between growth hormones and other pituitary factors in the modulation of depressive symptoms. Furthermore, they also point to a potential therapeutic role for changes in growth hormone in the amelioration of depression.

Together the studies included in this special issue contribute to our understanding of a continuously growing body of literature on the intersection of hormones and peptides in the brain and their influence on behaviors. We hope that this issue will further stimulate the studies necessary to help us better understand the connection between neuropeptides, hormones, and behavior.

## Author Contributions

SZ, JP, and JD conceived and edited the manuscript. SZ and JD wrote the manuscript. All authors contributed to the manuscript and approved the submitted version.

## Conflict of Interest

The authors declare that the research was conducted in the absence of any commercial or financial relationships that could be construed as a potential conflict of interest.

## Publisher's Note

All claims expressed in this article are solely those of the authors and do not necessarily represent those of their affiliated organizations, or those of the publisher, the editors and the reviewers. Any product that may be evaluated in this article, or claim that may be made by its manufacturer, is not guaranteed or endorsed by the publisher.
